# Arbovirus Seroprevalence Study in Bangphae District, Ratchaburi Province, Thailand: Comparison between ELISA and a Multiplex Rapid Diagnostic Test (Chembio DPP^®^ ZCD IgG)

**DOI:** 10.3390/tropicalmed7110378

**Published:** 2022-11-15

**Authors:** Ruba Chakma, Pimolpachr Sriburin, Pichamon Sittikul, Jittraporn Rattanamahaphoom, Warisa Nuprasert, Nipa Thammasonthijarern, Pannamas Maneekan, Janjira Thaipadungpanit, Watcharee Arunsodsai, Chukiat Sirivichayakul, Kriengsak Limkittikul, Supawat Chatchen

**Affiliations:** 1 Department of Tropical Pediatrics, Faculty of Tropical Medicine, Mahidol University, Bangkok 10400, Thailand; 2 Department of Parasitology, Faculty of Veterinary Medicine, Kasetsart University, Bangkok 10900, Thailand; 3 Department of Tropical Hygiene, Faculty of Tropical Medicine, Mahidol University, Bangkok 10400, Thailand; 4 Department of Clinical Tropical Medicine, Faculty of Tropical Medicine, Mahidol University, Bangkok 10400, Thailand; 5 Mahidol-Oxford Tropical Medicine Research Unit, Faculty of Tropical Medicine, Mahidol University, Bangkok 10400, Thailand

**Keywords:** dengue virus, zika virus, chikungunya virus, seroprevalence, ELISA, RDT, Thailand

## Abstract

Arboviruses, particularly dengue virus (DENV), Zika virus (ZIKV), and Chikungunya virus (CHIKV), pose a growing threat to global public health. For disease burden estimation and disease control, seroprevalence studies are paramount. This study was performed to determine the prevalence of DENV, ZIKV, and CHIKV on healthy individuals aged from 1–55 years old in Bangphae district, Ratchaburi province, Thailand. Enzyme-linked immunosorbent assays (ELISAs) and rapid diagnostic tests (RDTs) were performed on archived samples from a dengue serological survey conducted from 2012–2015. All 2012 samples had been previously tested using an anti-DENV immunoglobulin (Ig)G ELISA, and 400 randomly selected samples stratified by age, sex, and residential area were assessed by an in-house anti-ZIKV IgG ELISA and a commercial anti-CHIKV IgG ELISA to determine virus-specific antibody levels. An RDT (Chembio DPP^®^ ZCD IgM/IgG System) was also used to investigate the presence of antibodies against DENV, ZIKV, or CHIKV. The ELISA results indicate that the seroprevalences of DENV, ZIKV, and CHIKV were 84.3%, 58.0%, and 22.5%, respectively. The youngest age group had the lowest seroprevalence for all three arboviruses, and the seroprevalences for these viruses were progressively higher with increasing participant age. The DPP^®^ IgG sensitivities, as compared with ELISAs, for DENV, ZIKV, and CHIKV were relatively low, only 43.92%, 25.86%, and 37.78%, respectively. The ELISA results indicate that 16% of the study population was seropositive for all three viruses. DENV had the highest seroprevalence. ZIKV and CHIKV were also circulating in Bangphae district, Ratchaburi province, Thailand. The DPP^®^ ZCD rapid test is not sensitive enough for use in seroprevalence studies.

## 1. Introduction

Arboviruses are maintained in nature principally through their biological transmission between susceptible vertebrate hosts by blood-sucking arthropod vectors. These viruses are found worldwide but are more common in tropical and subtropical countries. There are ~500 known arboviruses, of which ~100 cause disease in humans and ~40 cause disease in domestic animals [1]. Although they are predominant in tropical and subtropical regions, owing to population growth and urbanization, international travel and trade, vector adaption, and global warming in recent decades, arbovirus infections have spread worldwide. Travelers have played a particularly important role in the worldwide transmission of mosquito-borne viruses [2,3]. Three major arthropod-borne viruses, dengue virus (DENV), Zika virus (ZIKV), and Chikungunya virus (CHIKV), are becoming public health concerns because of the growing threat they pose to global health and socioeconomic development. 

DENV and ZIKV are mosquito-borne single-stranded RNA viruses in the genus *Flavivirus*, family *Flaviviridae*. Dengue, an infection caused by four antigenically distinct DENVs, has caused a huge disease burden in tropical and subtropical regions [4,5]. Due to a dramatic increase in DENV incidence over recent decades, it is estimated that there are now approximately 390 million DENV infections each year worldwide, with 500,000 cases of severe dengue requiring hospitalization and 20,000 deaths [6,7,8]. ZIKV was first isolated from a rhesus monkey in the Zika forest in 1947 [9], and the first case of human infection was reported from Nigeria in 1954 [10]. The first outbreak of ZIKV infection was reported from the Yap islands and Federal states of Micronesia in 2007 [11]. The pandemic of ZIKV in Brazil began in Bahia, a northeastern state, and became a large outbreak throughout the Americas [12]. More than 3000 cases of microcephaly were identified, and ZIKV was isolated from the autopsy brain tissue from a ZIKV infected infant [13]. On February 2016, the World Health Organization declared ZIKV infection a public health emergency of international concern [14]. 

CHIKV belongs to the genus *Alphavirus* in the family *Togaviridae*. The primary vectors of this virus are the *Ades aegypti* and *Aedes albopictus* mosquitoes, the same vectors that spread DENV and ZIKV. The main symptoms of CHIKV infection are high fever and joint pain in the acute phase, often accompanied by headache, diffuse back pain, myalgia, nausea, vomiting, polyarthritis, rash, and conjunctivitis; these symptoms are similar to those observed in dengue fever, especially in the acute phase. The first clinical report of CHIKV fever was as early as the 1770s, and the first CHIKV outbreak in Asia was reported from Bangkok in 1958 [15]. CHIKV epidemics later occurred in Cambodia, Vietnam, Malaysia, and Taiwan. In the 2005–2006 outbreak of CHIKV in La Reunion Island in the Indian Ocean, infected patients presented with severe complicated manifestations, primarily associated with encephalopathy and hemorrhagic fever [16]. Owing to challenges in accurately diagnosing the disease, there is no accurate estimate for the number of people affected by this virus. Because of the severe debilitating nature of the disease and the discovery of a new mutant in Caribbean countries and territories in 2013, CHIKV has become a global threat [17].

In Thailand, which has a well-established national dengue surveillance system, the prevalence and incidence rates of dengue are documented. However, similar to other countries in Southeast Asia, information regarding the periodic outbreaks of other mosquito-borne viral infections, such as those caused by ZIKV or CHIKV, is scarce [18,19]. Moreover, the acute stages of arboviral infections usually cause undifferentiated clinical manifestations, ranging from asymptomatic to severe illness, such that these diseases cannot be accurately differentiated from each other [20,21]. Despite the evidence that the arboviral diseases caused by DENV, ZIKV, and CHIKV contribute substantially to morbidity in Thailand, there are only a few studies that investigated the seroprevalence of these three major arboviruses [22]. A regular confirmatory laboratory test for arbovirus infection is generally not performed in healthy or mildly ill individuals. Consequently, the actual disease burdens and their spreading natures cannot be accurately estimated and the distributions of these viruses in Thailand remain uncertain. 

A seroprevalence study can provide important information for disease control, and the development of a rapid diagnostic test (RDT) for use in such studies would ensure that low- and middle-income countries have a diagnostic tool for clinical management and surveillance, providing early alert against future outbreaks. The Chembio Dual Path Platform (DPP) ZCD (Zika/Chikungunya/Dengue) immunoglobulin (Ig)M/IgG system is a rapid immunochromatographic test for the separation and detection of IgM and IgG antibodies against DENV, ZIKV, and CHIKV in 10 µL of whole blood, serum, or plasma. Its results are read 10–15 min after adding buffer. Here, we investigated the seroprevalences of DENV, ZIKV, and CHIKV by testing archived samples from a dengue cohort study conducted in children and adults of central Thailand from 2012 to 2015 [23]. The present work aimed to explore the seroprevalence of DENV, ZIKV, and CHIKV by using ELISAs and the DPP^®^ ZCD IgM/IgG System to detect antibodies against DENV, ZIKV, and CHIKV in archived samples from a dengue cohort study of children and adults in Bangphae district, Ratchaburi province, Thailand.

## 2. Materials and Methods

### 2.1. Study Site and Serum Samples

**Figure 1:**
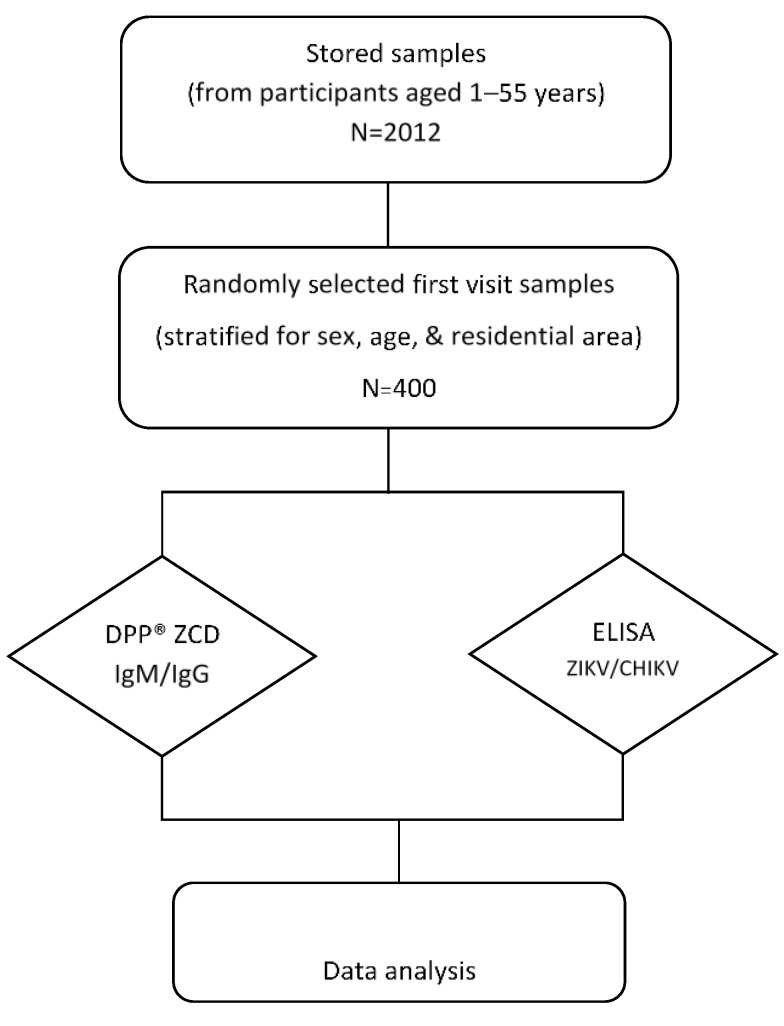
Schematic diagram of the study design and procedures. Four hundred serum samples were selected and used to perform ZIKV and CHIKV immunoglobulin (Ig) G enzyme-linked immunosorbent assay (ELISA) tests (DENV ELISA results were used from previous study) and the DPP^®^ ZCD IgM/IgG system.

This seroprevalence study was performed by using archived data and serum samples collected (stored at −80 °C) from a cohort study of DENV conducted in Bangphae district, Ratchaburi province, Thailand from 2012 to 2015 (Figure 1). This previous study enrolled 2012 healthy children and adults aged between 1 and 55 years in 2012. The subjects were prospectively followed for six visits during the period from 2012–2015. The longitudinal serosurvey study of DENV was performed using an indirect IgG ELISA against DENV as described previously [23]. It was found that the overall prevalence of past DENV infection as measured by IgG ELISA was 74.3% in 2012 and had increased to 79.4% by 2015. Because DENV, ZIKV, and CHIKV can be transmitted by the same mosquito vector and co-circulate in the same area, we selected the serum samples from the first visit in this Bangphae study for use in our research. The minimum sample size for the present arbovirus seroprevalence study was calculated from an estimated prevalence of 50% for ZIKV and CHIKV, with a 95% confidence level and 5% absolute precision. The estimated minimum sample size was then adjusted by 4% to allow for possible error, resulting in an estimated necessary sample size of approximately 400 samples. The DENV ELISA results were taken from the previous cohort study. All selected samples were assessed using ZIKV and CHIKV IgG ELISAs. The samples were also tested with the RDT (DPP^®^ ZCD IgM/IgG System). 

The protocol for this study was reviewed and approved by the Ethics Committee of the Faculty of Tropical Medicine, Mahidol University (protocol TMEC 22-016). Procedures conducted in this study were in accordance with the standard criteria of the Human Ethical Research committee of Faculty of Tropical Medicine, Mahidol University.

### 2.2. In-House DENV IgG ELISA

The present study used the in-house indirect sandwich DENV IgG ELISA using a dengue monoclonal antibody 2H2 and an equal mixture of inactivated DENV 1–4 antigen. The data from a previous report, in which the method used to acquire these data is described [23,24]. 

### 2.3. In-House ZIKV NS1 IgG ELISA

An in-house ZIKV NS1 IgG ELISA was performed using the ZIKV NS1 protein, a recombinant protein produced in *E. coli* BL21(DE3), to evaluate the immune status against ZIKV of the study participants [25,26]. Briefly, 96-well ELISA plates were sensitized with 60 µL/well of ZIKV NS1 protein (500 ng) in 0.018 M carbonate buffer. After an overnight incubation at 4 °C, the plates were washed six times with 300 µL/well of phosphate-buffered saline (PBS) containing 1% Tween 20 (PBS-T) and then blocked with assay buffer (5% skim milk in PBS-T) for 1 h at 37 °C. After additional washing procedures were conducted, 50 µL/well of the tested serum diluted 1:100 with assay buffer and controls were added in duplicate and then the plates were incubated at 37 °C for 1.5 h. After being washed again, the plates were filled with 50 µL/well of a goat anti-human IgG antibody conjugated to horse radish peroxidase (KPL Inc., Gaithersburg, MD, USA), diluted at 1:5000 in assay buffer. After being incubated at 37 °C for 90 min, the plates were washed six times, and the substrate SureBlue™ TMP (KPL Inc., Gaithersburg, MD, USA) was added for 30 min incubation at room temperature. The colorimetric reaction was stopped by the addition of 50 µL of 0.2 M sulfuric acid to each well, and the optical density (OD) at 450 nm was read using an ELISA plate reader. 

### 2.4. CHIKV IgG ELISA

The EUROIMMUN CHIKV ELISA test kit (EUROIMMUN Co., Lubeck, Germany) is a semi-quantitative in vitro assay for the detection of human IgG-class antibodies against CHIKV in serum. The kits contain microtiter strips, each with eight break-off reagent wells coated with CHIKV antigens. First, 10 µL of the sample was diluted into 1.0 mL of sample buffer. The Euroimmun anti-CHIKV IgG ELISA was performed as following the manufacturer’s instructions. The results were evaluated by calculating the ratio of the extinction value of the control or sample divided by the extinction value of the calibrator. This assay was evaluated on an ELISA plate reader. 

### 2.5. Chembio DPP^®^ ZCD IgM/IgG System

The DPP^®^ ZCD IgM/IgG System employs Chembio’s patented DPP technology (Dual membrane Path Platform), consisting of a sample path that distributes samples onto two test strips. The upper test strip (window labelled “1, 2, 3”) is for detecting IgM antibodies against DENV, ZIKV, and CHIKV, respectively, and the bottom test strip (window also labelled “1, 2, 3”) is for the detection of IgG antibodies against DENV, ZIKV, and CHIKV, respectively. To initiate the test, a 10-µL specimen is diluted with a buffer and applied to the SAMPLE + BUFFER well located in the middle of the sample transfer strip of the DPP^®^ ZCD IgM/IgG Test Device. The DPP^®^ ZCD IgM/IgG System was performed according to manufacturer’s instructions. At the time of reading the results, the DPP^®^ Micro reader and cassette adapter must be used to obtain the test results. 

### 2.6. Criteria for Seropositivity

For DENV and ZIKV IgG ELISA results, the criterium for seropositivity was the sample OD greater than or equal to twice over the OD from the negative control [23,25]. For the CHIKV ELISA results, a sample was classified as seropositive when the sample OD greater than or equal to 1.1 times over the OD from the calibrator. In accordance with the DPP^®^ ZCD IgM/IgG system protocol, a sample was classified as positive when the level of the sample from the reader was greater than or equal 22. The DPP^®^ ZCD IgG levels were used to compare with the ELISA results.

### 2.7. Statistical Analysis

A descriptive summary of study participant characteristics is presented stratified by sex, location, and age group; participant age was divided into five age groups for comparative purposes. The Mann-Whitney U-test was performed to compare continuous variables between study groups. The correlation coefficient was used to compare the DPP^®^ and ELISA results. Sensitivity, specificity, and predictive values were calculated for the DPP^®^ using the corresponding ELISA as a standard. Differences were considered statistically significant if the *p*-value was less than 0.05. All analyses were performed using SPSS software version 18.0 (SPSS Inc., Chicago, IL, USA).

## 3. Results

### 3.1. Demographic Data 

The demographic data for our study participants were described previously [23]. The serosurvey of dengue in Bangphae district, Ratchaburi province, Thailand from 2012 to 2015 was conducted by performing an indirect DENV IgG ELISA. Over that study period, approximately 2000 children and adults were enrolled in the dengue serosurvey. Archived serum samples from visit 1 (Y2012) and the associated demographic data were used for the present study. From each of the five age groups, a group of 80 serum samples with an equal sex and location distribution was randomly selected (total: 400 serum samples) for use in this study (Table 1).

### 3.2. Arbovirus Seroprevalences 

The seroprevalence of DENV, ZIKV, and CHIKV according to the ELISA results and a descriptive summary of participant characteristics are presented in Table 2. The IgG ELISA results show that the arbovirus with the highest seroprevalence was DENV (84.25%), followed by ZIKV (58.00%) and CHIKV (22.50%). According to the ELISA results, the DENV seroprevalence was very high in all tested areas (77.19–92.98%). 

Among the three arboviruses, DENV had the highest seroprevalence, according to the ELISA results, in all sub-districts (77.19–92.98%), while CHIKV had the lowest seroprevalence, particularly in the youngest three age groups. The pattern of seroprevalence for the three tested arboviruses varied in different areas. Wang-Yen sub-district had the highest percentage of DENV seropositivity (92.98%), Pho-Hak had the highest percentage of ZIKV seropositivity (80.70%), and Hau-Pho had the highest percentage of CHIKV seropositivity (35.09%). Don-Kha and Don-Yai had the lowest percentage of ZIKV seropositivity (40.35%), and Wat-Kaew had the lowest percentage of CHIKV seropositivity (14.04%).

**Figure 2:**
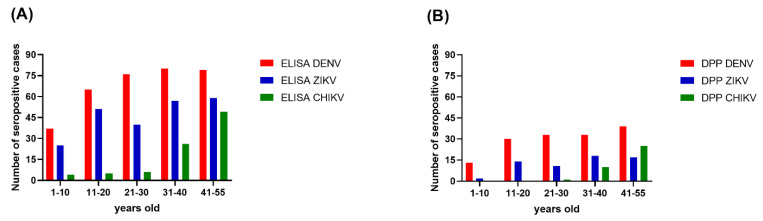
Seroprevalence of three arboviruses by age group. (**A**) The ELISA results; (**B**) The DPP^®^ ZCD IgG results. The x-axes describe the DENV (red), ZIKV (blue), and CHIKV (green) distributions for five age groups in Bangphae district, Ratchaburi province, Thailand. The y-axes represent the seroprevalence, as shown by the number of cases.

Overall, the seroprevalence for each of the three arboviruses was progressively higher with increasing age. The age-specific seroprevalence of DENV reached its peak level in those aged 31–40 years old. For ZIKV, the seroprevalence dropped from 63.75% in the 11–20 years old age group to 50% in the 21–30 years old age group, but trended upward again in the remaining older age groups (Figure 2). For CHIKV, the seroprevalence was 5% in the 1–10 years old age group, 6.25% in the 11–20 years old age group, and 7.50% in the 21–30 years old age group. The seroprevalence of CHIKV was dramatically higher in the 31–40 years old age group (32.5%) and higher again in the 40–55 years old age group (61.25%).

**Figure 3:**
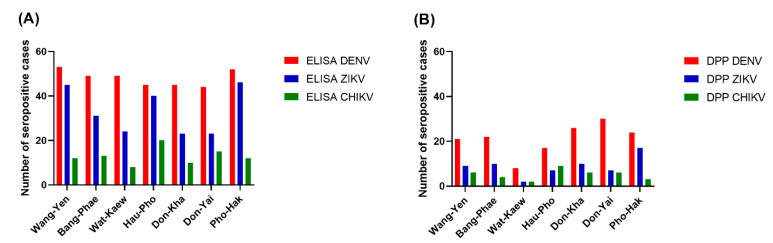
Seroprevalence of three arboviruses by residential area. (**A**) The ELISA results; (**B**) The DPP^®^ ZCD IgG results. The x-axes describe the DENV (red), ZIKV (blue), and CHIKV (green) distributions for the seven sub-districts of Bangphae district, Ratchaburi province, Thailand. The y-axes represent the seroprevalence, as shown by the number of cases.

The Chembio DPP^®^ ZCD results indicate overall DENV, ZIKV, and CHIKV seroprevalences lower than those estimated from the ELISA results. There were 160, 64, and 37 cases determined by the Chembio DPP^®^ ZCD as being seropositive for anti-DENV, -ZIKV, and -CHIKV IgM or IgG, respectively; the numbers of cases seropositive for only virus-specific IgG were 148, 62, and 36, respectively (Table 3). When judging the seroprevalence by the DPP data instead of the ELISA data, the areas with the highest seroprevalence of DENV and ZIKV were Don-Yai instead of Wang-Yen and Don-Kha instead of Pho-Hak, respectively, but the area with the highest seroprevalence of CHIKV (Hau-Pho) remained the same (Figure 3). The pattern of age-specific seroprevalence for these arboviruses in children and adults according to the DPP data was similar to those estimated from the ELISA data but with lower seroprevalences (Table 3, Figure 2). 

### 3.3. Co-Seropositivity for DENV, ZIKV, and CHIKV 

**Figure 4:**
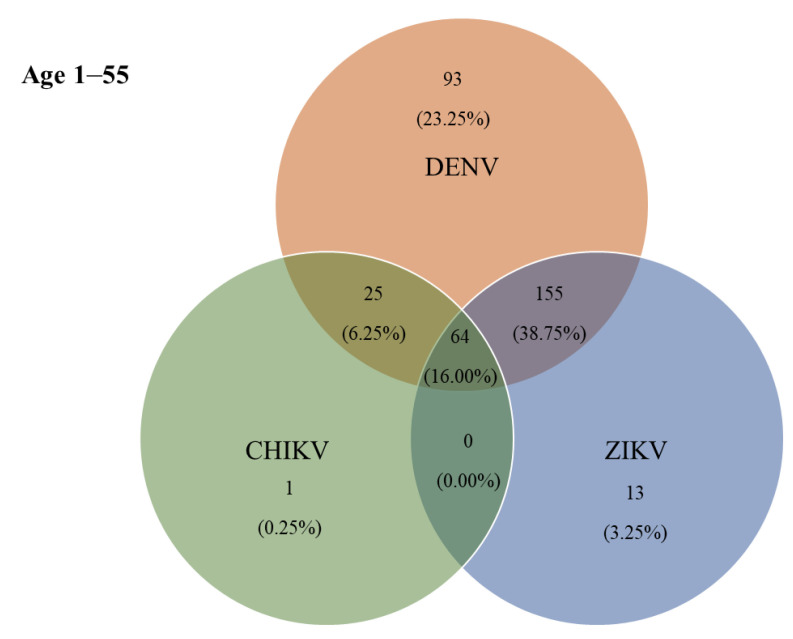
Co-circulation of three ELISA-positive arboviruses in Bangphae district, Ratchaburi province, Thailand. Number and percentage (%) of samples found to be positive by ELISA for immunoglobulin G (IgG) antibodies to single or multiple exposures of DENV, ZIKV, and CHIKV.

According to the ELISA results, 115 (38.75%) individuals were jointly positive for DENV- and ZIKV-specific IgG antibodies, and 64 (16.00%) were jointly positive for DENV-, ZIKV-, and CHIKV-specific IgG antibodies (Figure 4). The prevalence of IgG antibodies against all three arboviruses (DENV, ZIKV, and CHIKV) was highest in Hau-Pho sub-district (18 cases), followed by Bang-Phae sub-district (12 cases), and Pho-Hak sub-district (9 cases). The age group with the highest joint seroprevalence of DENV and ZIKV was the 11–20-years-old group at 52.50%, whereas the highest joint seropositivity for DENV-specific and CHIKV-specific antibodies was 15.00% in the 41–55-years-old age group. The prevalence of IgG antibodies against all three arboviruses showed a gradual rise with age during early life (i.e., slightly higher prevalence in young adults than in children); however, the prevalence of these antibodies rapidly became higher with increasing ages above 30 years (Table 4). The highest co-seropositivity (38.75%) was seen for DENV-specific and ZIKV-specific antibodies. In contrast, only 25 (6.25%) of study participants were jointly seropositive for DENV-specific and CHIKV-specific antibodies. The Chembio DPP^®^ IgG revealed 87 (21.75%) individuals as seropositive for DENV-specific IgG, 14 (3.50%) for ZIKV-specific IgG, and 10 (2.50%) for CHIKV-specific IgG. Moreover, the co-seropositive for both DENV and ZIKV IgG, and for DENV, ZIKV, and CHIKV IgG were 35 (8.75%) and 13 (3.25%), respectively.

### 3.4. Comparison of ELISA and Chembio DPP^®^ ZCD IgG 

Among the three arboviruses, DENV had the highest seroprevalence, according to both the ELISA and Chembio DPP^®^ ZCD system results. Comparing the ELISA and Chembio DPP^®^ ZCD IgG results revealed that the sensitivity of the Chembio DPP^®^ IgG for DENV was relatively low, only 43.92% (95% confidence interval (CI): 38.54–49.40%). Additionally, although the specificity was 100% (95% CI: 94.31–100.00%), the accuracy was only 52.75% (95% CI: 47.73–57.73%). The sensitivity and specificity of the Chembio DPP^®^ IgG for ZIKV as compared with ELISA were 25.86% (95% CI 20.35–32.00%) and 98.81% (95% CI: 95.77–99.86%), respectively; the accuracy was only 56.50% (95% CI: 51.48–61.42%). The accuracy for CHIKV rapid tests (85.50%, 95% CI: 81.66–88.80%) was relatively higher compared with the rapid tests for DENV and ZIKV; however, the sensitivity was only 37.78% (95% CI: 27.77–48.62%), and the specificity was 99.35% (95% CI: 97.69–99.92%).

**Figure 5:**
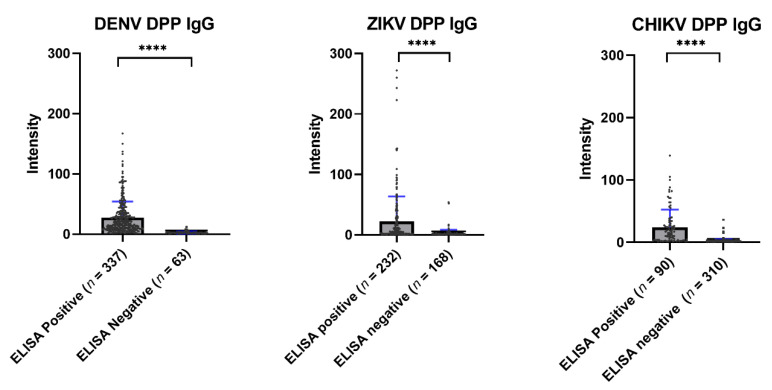
Scatter plot of the DPP^®^ ZCD system results by ELISA-positive and ELISA-negative samples. The median DENV DPP^®^ results from ELISA-positive and -negative samples were 18.00 and 2.50, respectively (**** *p* < 0.0001). The median ZIKV DPP^®^ results from ELISA-positive and -negative samples were 5.90 and 1.30, respectively (**** *p* < 0.0001). The median CHIKV DPP^®^ results from ELISA-positive and -negative samples were 14.50 and 2.10, respectively (**** *p* < 0.0001).

Overall, the immunochromatographic test of the Chembio DPP^®^ ZCD system showed a low sensitivity when compared with ELISA. Figure 5 shows the average values of the Chembio DPP^®^ ZCD IgG in the ELISA-based seropositive and seronegative groups. All Chembio DPP^®^ ZCD systems for the three arboviruses produced a statistically significant difference in antibody levels between the ELISA-based seropositive and seronegative groups.

## 4. Discussion

The primary purpose of this study was to assess the seroprevalence status of three important arboviruses, i.e., DENV, ZIKV, and CHIKV. In addition, this study also aimed to evaluate the sensitivity and specificity of the DPP^®^ ZCD IgG rapid test as compared with ELISA and assess the prevalence of exposure to multiple arboviruses in the population. To this end, 400 serum samples, from healthy participants without febrile illness evenly split across five age groups with equal male:female ratios and equal representation of seven sub-districts of the Bangphae district, Ratchaburi province, Thailand, were selected from archived serum samples collected by the previous study, “Dengue virus seroprevalence study in Bangphae district, Ratchaburi, Thailand: A cohort study in 2012–2015” [23]. 

The seroprevalence of exposure to all three major arboviruses was progressively higher with advancing age, i.e., the younger age groups had lower seroprevalences compared with the older age groups. This occurrence in endemic areas may be due to older people having a higher cumulative exposure to these arboviruses. From the ELISA results, the highest percentage of co-seropositivity was for the combination of DENV and ZIKV (38.75%). DENV and ZIKV are closely related flaviviruses that share a high degree of structural and sequence homology. Moreover, the cross-reactivity between DENV and ZIKV is a challenging serological diagnostic problem. The cross-reactivity of antibodies between these two closely related viruses could explain this finding. A previous study on the seroprevalence of Zika in pregnant women in Thailand also noticed a cross-reaction between DENV and ZIKV [27]. According to past studies, the sensitivity and specificity of in-house DENV IgG ELISA were 94.37% and 87.33%, respectively, when comparing with PRNT [24]. Comparison with PRNT results revealed that the sensitivity and the specificity of the in-house ZIKV NS1 IgG ELISA were 100% and 70.27%, respectively [26]. Furthermore, this low specificity in the ZIKV NS1 IgG ELISA mainly caused by the secondary DENV infection samples. Additionally, the trend of ZIKV seroprevalence differed across age groups, which could be the result of susceptibility variation, as suggested by a previous study on ZIKV that also proposed variable susceptibility to ZIKV [28]. Other related work found that age and sex had roles in susceptibility to ZIKV infection [29]. In the present study, the arbovirus with the highest seroprevalence was found to be DENV in all seven sub-districts, followed by ZIKV and CHIKV. Compared with other sub-districts, Wang-Yen and Pho-Hak had the highest seroprevalence of DENV and ZIKV, respectively, and Hua-Pho had the highest seroprevalence of CHIKV. A previous CHIKV study in Vietnam found a different scenario for CHIKV seroprevalence, reporting that different areas had different patterns [15]. Here, the younger age group had little exposure to CHIKV, and the seroprevalence of this virus was progressively higher among the middle and older age groups but varied among different locations. This study result is similar to that of a 2017 study on CHIKV seroprevalence in India, in which the younger age group had a lower seroprevalence compared with the older age group, and the seroprevalence was progressively higher in increasingly older groups [30].

Our work reveals the co-circulation of three important arboviruses in the study area. DENV had the highest seroprevalence among the tested arboviruses. The ELISA result indicates that there was no statistically significant difference in seropositivity for DENV, ZIKV, or CHIKV between males and females, although the percentages of seropositivity for DENV and CHIKV trended higher in the group of female participants and the percentage of seropositivity for ZIKV trended higher in the group of male participants. The similarity of the DENV seroprevalence found in our work to that reported by a previous study confirms the appropriate sampling of serum samples in this research [23]. Regarding ZIKV, our finding that the seroprevalence trended slightly higher in male participants than in female participants is inconsistent with the report by a previous ZIKV study in Thailand showing 61% of confirmed ZIKA cases occurring in female patients [31,32]. As ZIKV infection can impact pregnancy outcome, infection of female individuals with this virus remains a major concern. Regarding CHIKV, the observed trend of a higher seroprevalence in female participants than in male participants in our study is similar to the reported higher percentage of CHIKV-confirmed cases in female patients than in male patients (57.8% vs. 42.2%) during a large-scale outbreak of CHIKV infection in Thailand in 2018–2019 [33]. However, an opposite scenario was seen in a previous study of CHIKV conducted in southern and central Vietnam [15]. Regarding the secondary study objective of comparing DPP^®^ ZCD with ELISA, we found that although the specificity of DPP^®^ ZCD was high, its sensitivity and accuracy were relatively low; a Kappa test showed a positive correlation between the ELISA and DPP^®^ rapid test results. Higher ELISA values were positively correlated with higher DPP^®^ values. The previous study on the evaluation of Zika rapid tests found that these tests had relatively higher sensitivity for clinical diagnosis of ZIKV [34]. However, the sensitivity of the ZIKV rapid test from the present serosurvey study was low compared with the sensitivity results for DENV and CHIKV rapid tests. The accuracy of the CHIKV rapid test was 85.50%, which is the highest accuracy value among all three rapid tests in the DPP^®^ ZCD system.

The limitations of the study were also considered. The study samples were selected from previously archived (10 years ago) serum collected in a dengue endemic area, which lacked information regarding ZIKV and CHIKV infections. Moreover, the ELISAs for the DENV-specific and ZIKV-specific antibody were performed with in-house ELISAs, whereas a commercial ELISA was used for the detection of the CHIKV-specific antibody. 

## 5. Conclusions

This study shows that, among DENV, ZIKV, and CHIKV, DENV had the highest seroprevalence; however, ZIKV and CHIKV are still important co-circulating arboviruses in Bangphae district, Ratchaburi province, Thailand. The DPP^®^ ZCD rapid test alone may not be practical for use in a seroprevalence study, owing to its low sensitivity compared with ELISAs. Further study to determine the true scenario of arbovirus disease burden and their spreading nature is needed in other endemic areas for informing future disease prevention and control.

## Figures and Tables

**Table 1:** Demographic characteristic of subjects [*n* (%)].

	Visit 1	Selected
**number/samples**	2012	400
**Male:Female**	903:1109	200:200
	0.81:1	1:1
**Sub-district**		
Wang-Yen	348 (17.30)	57
Bang-Phae	301 (15.00)	58
Wat-Kaew	250 (12.40)	57
Hau-Pho	254 (12.60)	57
Don-Kha	152 (7.60)	57
Don-Yai	203 (10.00)	57
Pho-Hak	504 (25.00)	57
**Age group**		
1–10	695 (34.54)	80
11–20	733 (36.43)	80
21–30	180 (8.95)	80
31–40	170 (8.45)	80
41–55	234 (11.63)	80

**Table 2:** Seropositivity to three arboviruses according to ELISA [*n* (%)].

	DENV	ZIKV	CHIKV
**number/samples**	337 (84.25)	232 (58.00)	90 (22.50)
**Male:Female**	163:174	117:115	39:51
	0.94:1	1.02:1	0.76:1
**Sub-district**			
Wang-Yen	53 (92.98)	45 (78.95)	12 (21.05)
Bang-Phae	49 (84.48)	31 (53.45)	13 (22.41)
Wat-Kaew	49 (85.96)	24 (42.11)	8 (14.04)
Hau-Pho	45 (78.95)	40 (70.18)	20 (35.09)
Don-Kha	45 (78.95)	23 (40.35)	10 (17.54)
Don-Yai	44 (77.19)	23 (40.35)	15 (26.32)
Pho-Hak	52 (91.23)	46 (80.70)	12 (21.05)
**Age group**			
1–10	37 (46.25)	25 (31.25)	4 (5.00)
11–20	65 (81.25)	51 (63.75)	5 (6.25)
21–30	76 (95.00)	40 (50.00)	6 (7.50)
31–40	80 (100.00)	57 (71.25)	26 (32.50)
41–55	79 (98.75)	59 (73.75)	49 (61.25)

**Table 3:** Seropositivity for three arboviruses according to DPP^®^ ZCD system [*n* (%)].

	DENV IgM	DENV IgG	ZIKV IgM	ZIKV IgG	CHIKV IgM	CHIKV IgG
**number/** **samples**	28 (7.00)	148 (37.00)	5 (1.25)	62 (15.50)	14 (3.50)	35 (8.75)
**Male:Female**	17:11	72:76	3:2	30:32	6:8	14:21
	1.55:1	0.95:1	1.50:1	0.94:1	0.75:1	0.67:1
**Sub-district**						
Wang-Yen	4 (7.02)	21 (36.84)	1 (1.75)	9 (15.79)	2 (3.51)	6 (12.53)
Bang-Phae	2 (3.45)	22 (37.93)	0 (0.00)	10 (17.24)	2 (3.45)	4 (6.90)
Wat-Kaew	1 (1.75)	8 (14.04)	1 (1.75)	2 (3.51)	3 (5.26)	2 (3.51)
Hau-Pho	1 (1.75)	17 (29.82)	1 (1.75)	7 (12.28)	2 (3.51)	9 (15.79)
Don-Kha	3 (5.26)	26 (45.61)	1 (1.75)	10 (17.54)	1 (1.75)	5 (8.77)
Don-Yai	11 (19.30)	30 (52.63)	1 (1.75)	7 (12.28)	2 (3.51)	6 (10.53)
Pho-Hak	6 (10.53)	24 (42.11)	0 (0.00)	7 (12.28)	2 (3.51)	3 (5.26)
**Age group**						
1–10	3 (3.75)	13 (16.25)	1 (1.25)	2 (2.50)	4 (5.00)	0 (0.00)
11–20	6 (7.50)	30 (37.50)	2 (2.50)	14 (17.50)	2 (2.50)	0 (0.00)
21–30	8 (10.00)	33 (41.25)	1 (1.25)	11 (13.75)	3 (3.75)	1 (1.25)
31–40	5 (6.25)	33 (41.25)	1 (1.25)	18 (22.50)	2 (2.50)	10 (12.50)
41–55	6 (7.50)	39 (48.75)	0 (0.00)	17 (21.25)	3 (3.75)	24 (30.00)

**Table 4:** Co-circulation of three arboviruses according to ELISA [*n* (%)].

	DENV	ZIKV	CHIKV	D + Z	D + C	Z + C	D + Z + C
**number/** **samples**	93 (23.25)	13 (3.25)	1 (0.25)	155 (38.75)	25 (6.25)	0 (0.00)	64 (16.00)
**Age group**							
1–10	17 (21.25)	6 (7.50)	1 (1.25)	17 (21.25)	1 (1.25)	0 (0.00)	2 (2.50)
11–20	18 (22.50)	6 (7.50)	0 (0.00)	42 (52.50)	2 (2.50)	0 (0.00)	3 (3.75)
21–30	33 (41.25)	0 (0.00)	0 (0.00)	37 (46.25)	3 (3.75)	0 (0.00)	3 (3.75)
31–40	16 (20.00)	0 (0.00)	0 (0.00)	38 (47.50)	7 (8.75)	0 (0.00)	19 (23.75)
41–55	9 (11.25)	1 (1.25)	0 (0.00)	21 (26.25)	12 (15.00)	0 (0.00)	37 (46.25)

## Data Availability

Not applicable.
